# Frequency of Venous Thromboembolism in Patients with Liver Cirrhosis

**DOI:** 10.7759/cureus.9594

**Published:** 2020-08-06

**Authors:** Muhammad Omer Sultan, Umar Farooque, Muhammad Inam Khan, Sundas Karimi, Omer Cheema, Ali Jaan, Farhan Khalid, Muhammad Taimur, Fahham Asghar, Rafay Javed, Komal Girdhari

**Affiliations:** 1 Internal Medicine, Jinnah Postgraduate Medical Center, Karachi, PAK; 2 Neurology, Dow University of Health Sciences, Karachi, PAK; 3 General Surgery, Combined Military Hospital, Karachi, PAK; 4 Internal Medicine, Dow University of Health Sciences, Karachi, PAK; 5 Internal Medicine, King Edward Medical University, Mayo Hospital, Lahore, PAK; 6 Neurology, Dow Medical College, Dow University of Health Sciences, Karachi, PAK; 7 Internal Medicine, Jinnah Hospital, Allama Iqbal Medical College, Lahore, PAK; 8 Internal Medicine, Sukkur Civil Hospital, Sukkur, PAK

**Keywords:** venous thromboembolism, liver cirrhosis, humans, hypercoagulability, obesity, child-pugh class

## Abstract

Introduction

The major hemostatic problem in cirrhotic patients is the increased risk of bleeding, but venous thromboembolism is also being reported as a noticeable feature of cirrhosis. Therefore, we conducted this study to determine the frequency of venous thromboembolism in patients with liver cirrhosis.

Materials and methods

This cross-sectional study took place at a major metropolitan hospital in Karachi for a period of six months. A total of 142 patients age 40 to 70 years, either gender and Child-Pugh class A to C liver cirrhosis for >3 months were enrolled in this study. The demographic features like age, gender, weight, height, body mass index (BMI), duration of symptoms, and Child-Pugh class were noted. The patients were examined for calf swelling, tenderness, and pitting edema. Venous thrombosis was diagnosed on ultrasound of the calf done by an experienced radiologist in patients having two or more than two of the above-stated findings. The mean and standard deviation were calculated for age, weight, height, body mass index (BMI), and duration of symptoms. The frequency and percentage were calculated for the range of age, gender, range of weight, range of height, range of BMI, range of duration of symptoms, Child-Pugh class, and venous thrombosis. Stratification was done of venous thrombosis with age, obesity, gender, Child-Pugh class, and duration of symptoms by applying the chi-square test and assuming p-value ≤0.05 as significant.

Results

The mean age of the study population was 60.73 ± 10.83 years and most patients, i.e., 95 (66.9%) were >60 years. There were 89 (62.7%) female and 53 (37.3%) male patients. The mean weight of the study population was 60.15 ± 5.11 kg and most patients, i.e., 81 (57%), weighed ≤60 kg. The mean height of the study population was 1.53 ± 0.59 m and most patients, i.e., 99 (69.7%) were ≤1.5 m. The mean BMI of the study population was 27.24 ± 5.02 kg/m^2^ and most patients, i.e., 81 (57%) were ≤30 kg/m^2^. The mean duration of symptoms of the patients was 5.63 ± 1.77 months and most patients, i.e., 86 (60.6%) had ≤6 months of duration of symptoms. Eighty-six (60.56%) patients had Child-Pugh class A, 39 (27.47%) patients had Child-Pugh class B, and 17 (11.97%) patients had Child-Pugh class C liver cirrhosis. Ten (7%) of the patients had venous thrombosis. Stratification of venous thrombosis with age, gender, obesity, Child-Pugh class, and duration of symptoms showed a significant linear relationship with gender (p-value= 0.040), obesity (p-value= 0.043), and Child-Pugh class (p-value= 0.001).

Conclusions

Venous thromboembolism is a frequent complication and a pathogenic factor in liver cirrhosis that should be given attention to in cirrhotic patients especially in male and obese patients of Child-Pugh class B and C. Low serum albumin and increased partial thromboplastin time (PTT) can have some role in its prediction and early prevention. But more studies are needed to establish this.

## Introduction

Liver cirrhosis is not only associated with the decreased production of coagulation factors like factors II, VII, IX, and X, and decreased platelet count, but it also leads to decreased synthesis of anticoagulation factors such as protein C, protein S and anti-thrombin III. That’s why cirrhotic patients have not only the increased tendency of bleeding but some also present with venous thromboembolism [1-3]. But the frequency and risk of venous thromboembolism in cirrhotic patients as compared to the general population still needs to be determined as the available data is controversial.

One research done in the general population showed that cirrhotic patients were less prone to venous thromboembolism as compared to others (odds ratio (OR), 0.1; 95% CI, 0.0-0.7) [4].

Another research done in hospitalized patients also indicated that cirrhotic patients had a decreased risk of venous thromboembolism as compared to other admitted patients [5].

However, recent literature is pointing towards a higher risk of venous thromboembolism in admitted patients with cirrhosis (2.7-6.3%) [6-7]. Likewise, an analysis done at a metropolitan hospital, with 963 admitted cirrhotic patients and 12,405 others, established that cirrhotic patients were more likely to develop venous thromboembolism than other patients except those with congestive heart failure, chronic kidney disease, and cancer, who were at a higher risk of venous thromboembolism than cirrhotic patients but a multivariate analysis done in this study demonstrated no relationship between cirrhosis and venous thromboembolism (OR, 0.87; 95% CI, 0.28-2.63) [8].

These studies did not include cirrhotic patients admitted in intensive care units as all intensive care patients are more prone to venous thromboembolism, including those with cirrhosis [9].

The rationale of this study was that the magnitude of venous thromboembolism in cirrhotic patients varies from one study to another internationally and no local figures are available. Therefore, this study was designed with the intention to generate local data.

## Materials and methods

Study design and sampling

This cross-sectional study was conducted at Jinnah Postgraduate Medical Center, Karachi, from February 01, 2019, to July 30, 2019 (six months). The non-probability consecutive sampling technique was used. The sample size was calculated by assuming the frequency of venous thromboembolism in liver cirrhosis patients as 6.3%, confidence level at 95%, and margin of error at 4%. The input of the aforementioned information in Epi Info 7 (Centers for Disease Control and Prevention, Atlanta, Georgia) estimated the sample size of 142 patients. Inclusion criteria were age 40-70 years, either gender, and diagnosed liver cirrhosis for more than three months from Child-Pugh class A to C. Exclusion criteria included patients with venous thromboembolism prophylaxis, known active venous thromboembolism, palliative care, non-consenting, hepatocellular carcinoma, bacterial infection, and renal dysfunction.

Data collection

Cirrhotic patients meeting the inclusion criteria were selected for the study. All participants were educated about the study before inclusion and informed consent was taken. The principal investigator examined for tenderness in lower limbs, swelling of the calves (measured 10 cm below the tibial tuberosity and taken positive if >3 cm in circumference as compared to the normal calf) and thighs, and pitting edema in the affected limb. In the presence of any two or more of the above-stated symptoms, venous ultrasonography was done by radiologists with two or more than two years of experience and the presence of thrombus in the vein was labeled as venous thrombosis. Other demographics like age, gender, weight, height, body mass index (BMI), duration of symptoms, and Child-Pugh class were also noted.

Data analysis

Data were entered and analyzed on SPSS version 19 (SPSS Statistics, Chicago, IL). Mean and standard deviation was calculated for continuous variables such as age, weight, height, BMI, and duration of symptoms. Frequency and percentage were calculated for categorical variables such as range of age, gender, range of weight, range of height, range of BMI, range of duration of symptoms, Child-Pugh class, and venous thrombosis. Stratification of age, obesity, gender, Child-Pugh class, and duration of symptoms was done, the chi-square test was applied, and p-value ≤0.05 was considered significant.

## Results

The mean age of the patients was 60.73 ± 10.83 years, as shown in Table 1.

**Table 1:** Analysis of age

Age (years)	Mean ± standard deviation	Minimum	Maximum
	60.73 ± 10.83	45	70

**Figure 1:**
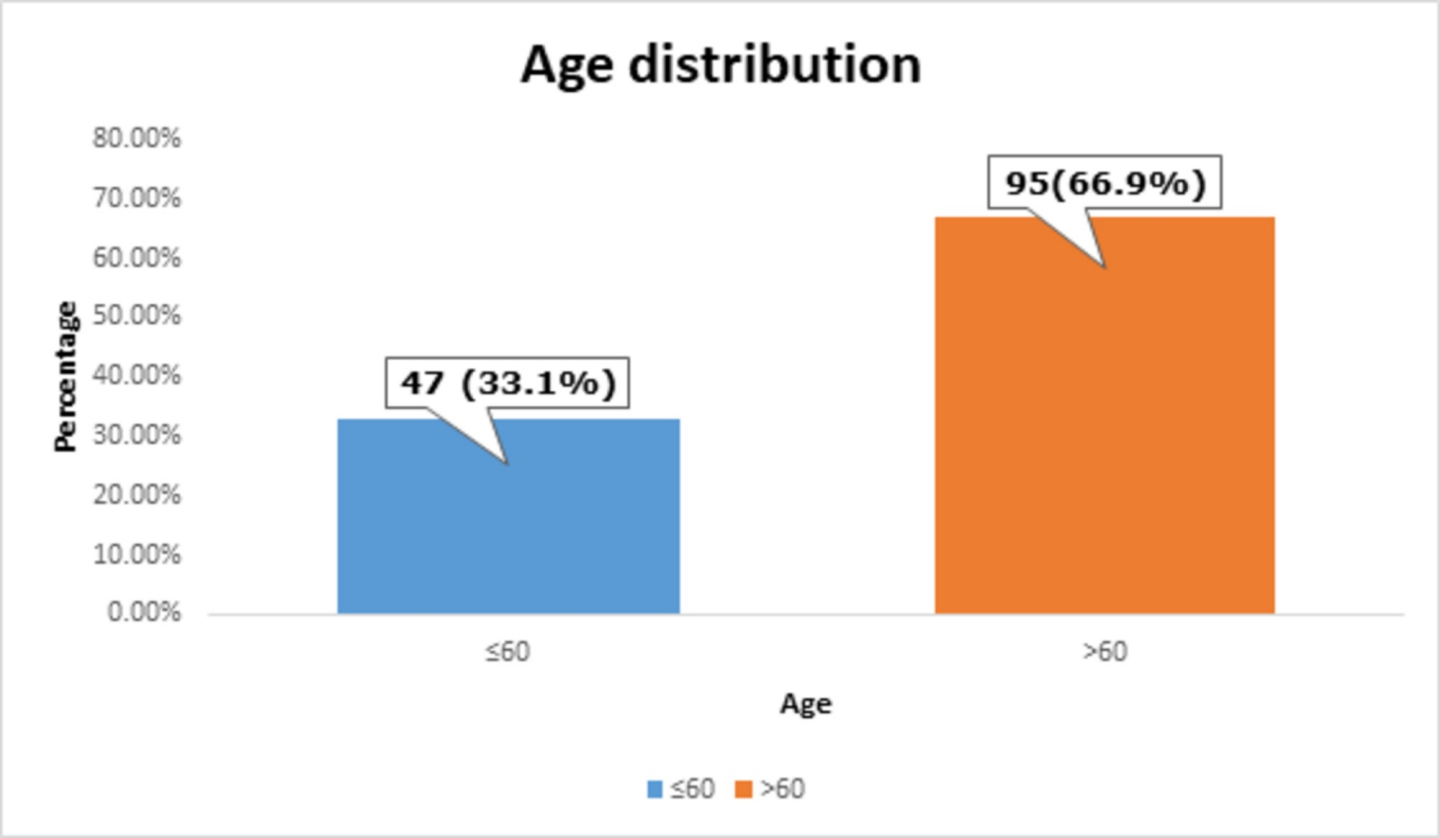
Age distribution

Most of the patients, i.e., 95 (66.9%) had >60 years of age, as shown in Figure 1.

**Figure 2:**
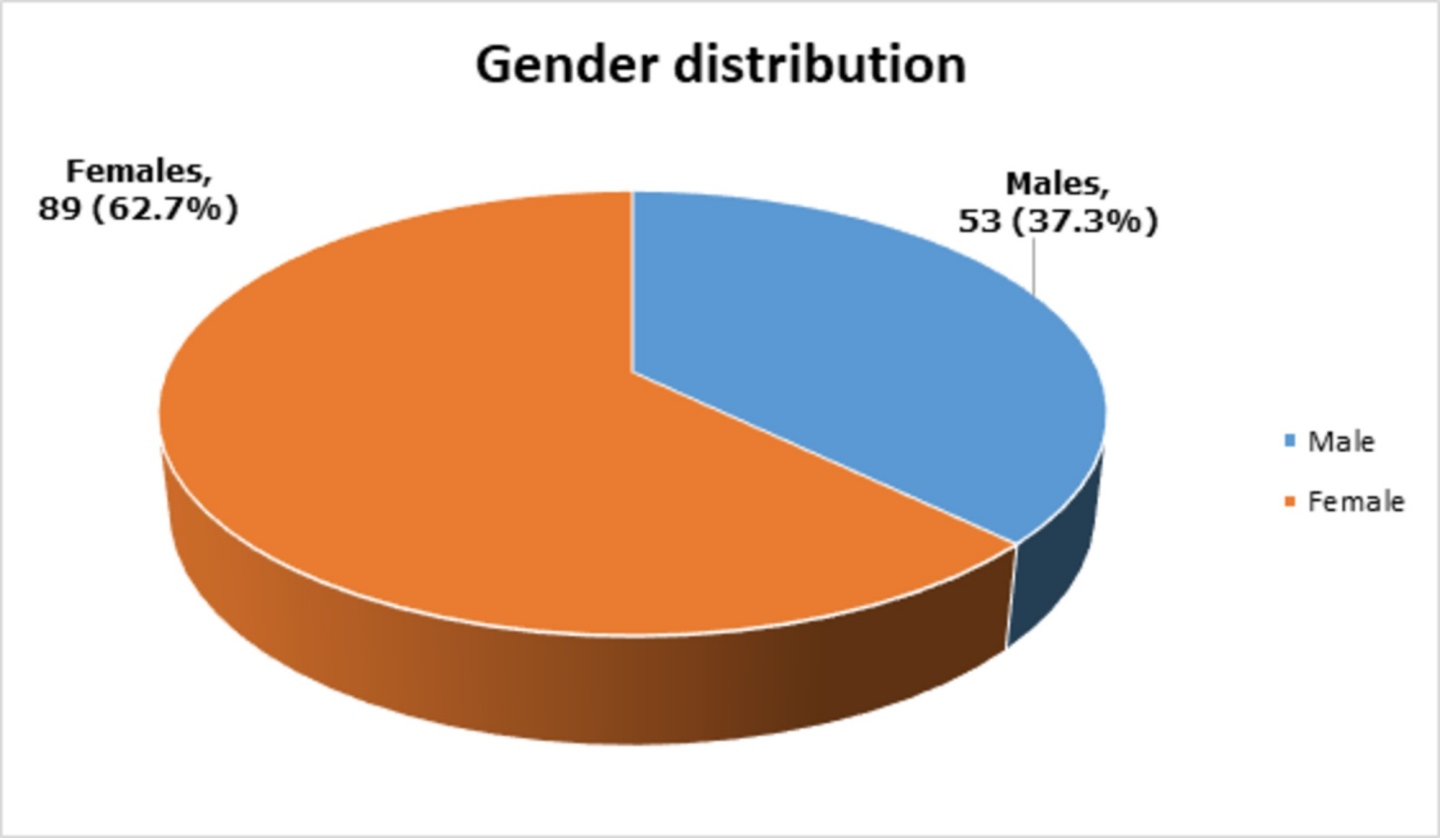
Gender distribution

There were 89 (62.7%) females and 53 (37.3%) males, as shown in Figure 2.

The mean weight of the patients was 60.15 ± 5.11 kg, as shown in Table 2.

**Table 2:** Analysis of weight

Weight (kg)	Mean ± standard deviation	Minimum	Maximum
	60.15 ± 5.11	53	66

**Figure 3:**
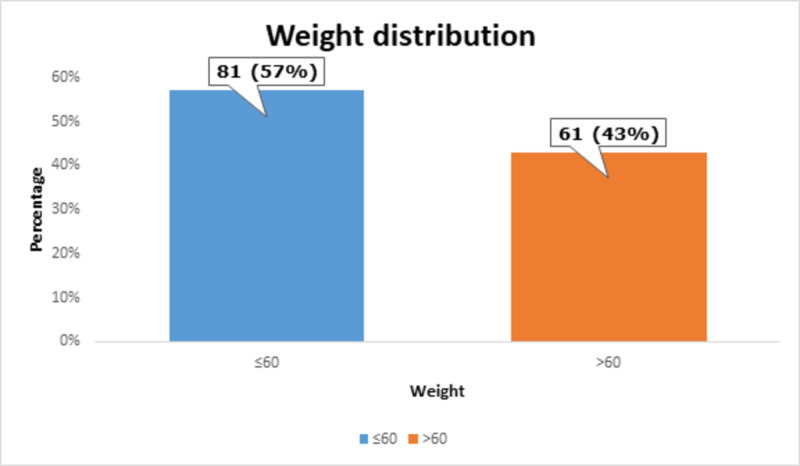
Weight distribution

Most of the patients, i.e., 81 (57%) had ≤60 kg of weight, as shown in Figure 3.

The mean height of the patients was 1.53 ± 0.59 m, as shown in Table 3.

**Table 3:** Analysis of height

Height (m)	Mean ± standard deviation	Minimum	Maximum
	1.53 ± 0.59	1.50	1.63

**Figure 4:**
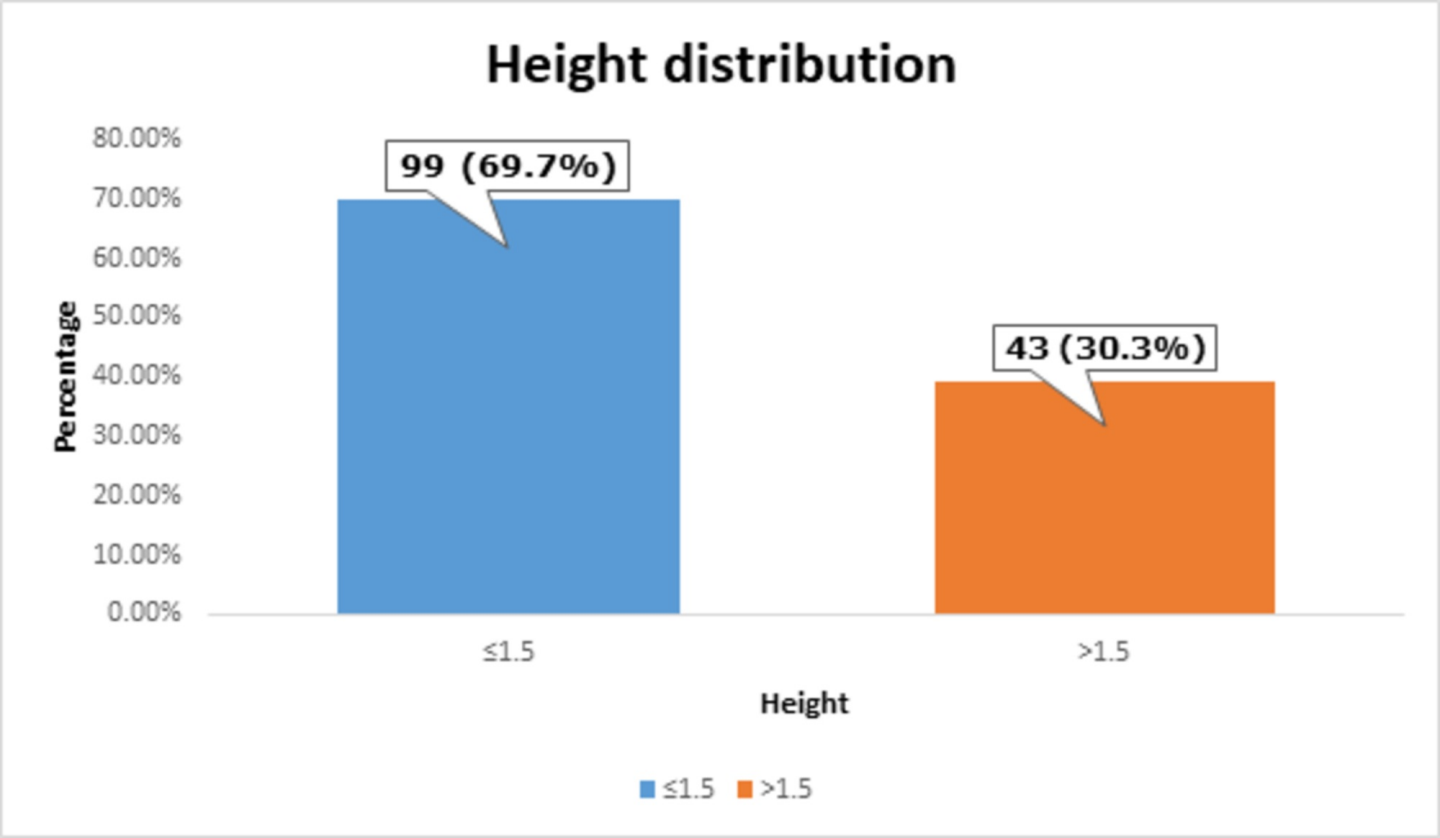
Height distribution

Most of the patients, i.e., 99 (69.7%) had ≤1.5 m of height, as shown in Figure 4.

The mean BMI of the patients was 27.24 ± 5.02 kg/m^2^, as shown in Table 4.

**Table 4:** Analysis of BMI BMI: body mass index

BMI (kg/m^2^)	Mean ± standard deviation	Minimum	Maximum
	27.24 ± 5.02	18.70	33.14

**Figure 5:**
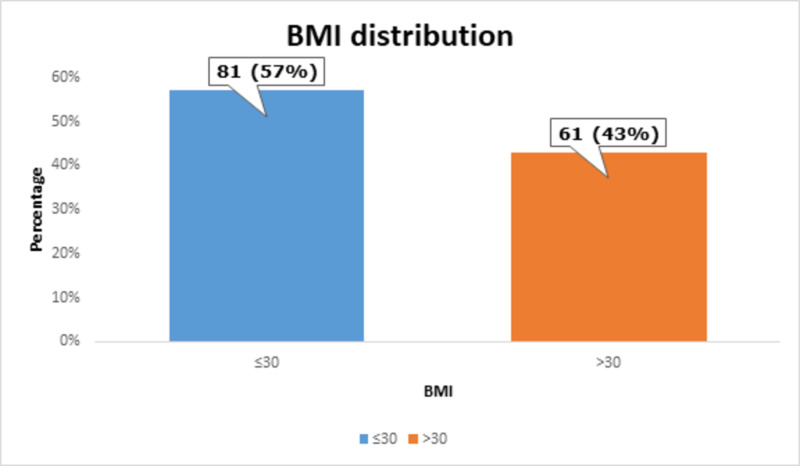
BMI distribution BMI: body mass index

Most of the patients, i.e., 81 (57%) had ≤30 kg/m^2^ of BMI, as shown in Figure 5.

The mean duration of symptoms was 5.63 ± 1.77 months, as shown in Table 5.

**Table 5:** Analysis of duration of symptoms

Duration of symptoms (months)	Mean ± standard deviation	Minimum	Maximum
	5.63 ± 1.77	4	8

**Figure 6:**
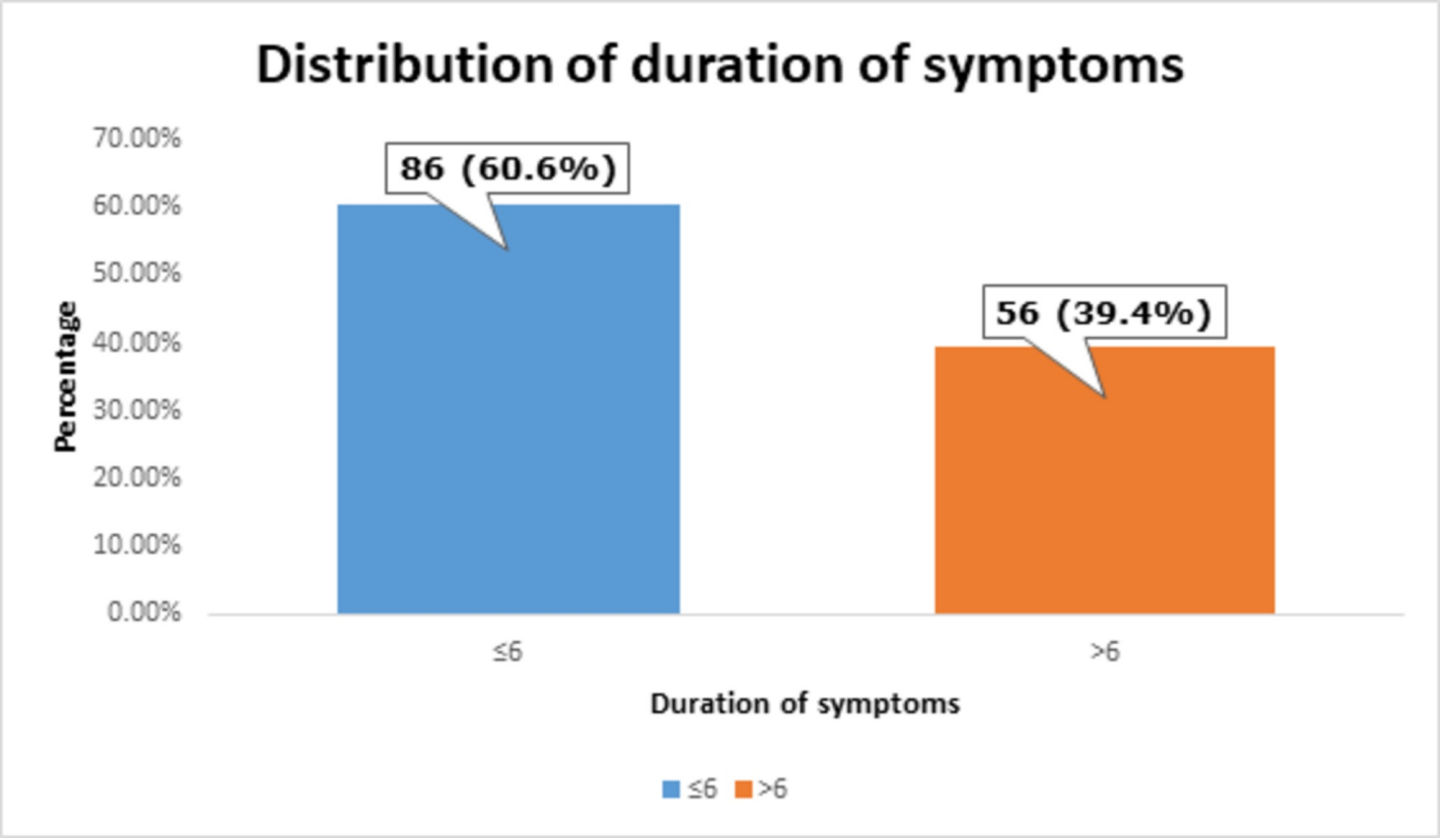
Distribution of duration of symptoms

Most of the patients, i.e., 86 (60.6%) had ≤6 months of duration of symptoms, as shown in Figure 6.

**Figure 7:**
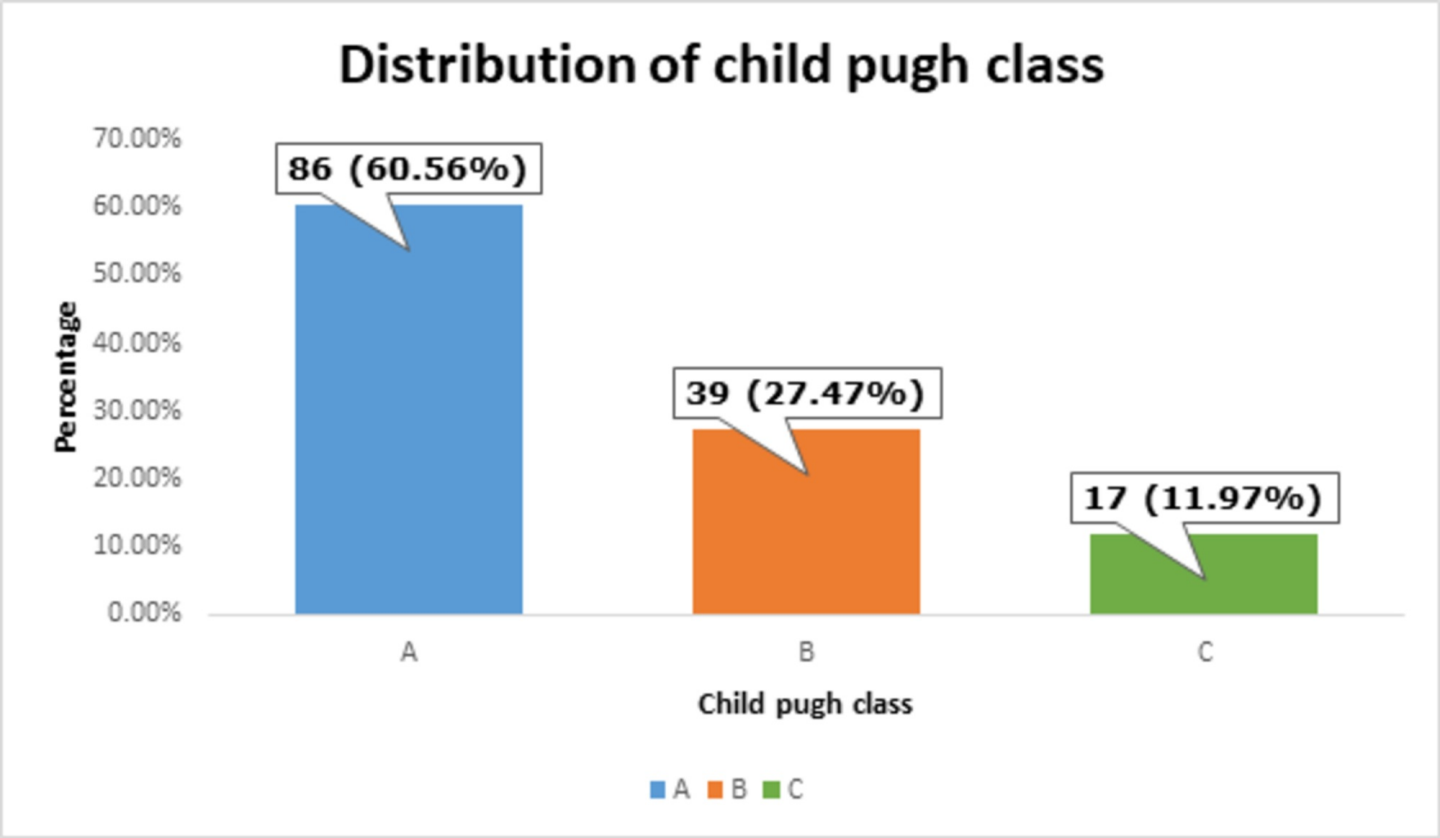
Distribution of Child-Pugh class

There were 86 (60.56%) Child-Pugh class A, 39 (27.47%) Child-Pugh class B, and 17 (11.97%) Child-Pugh class C patients, as shown in Figure 7.

**Figure 8:**
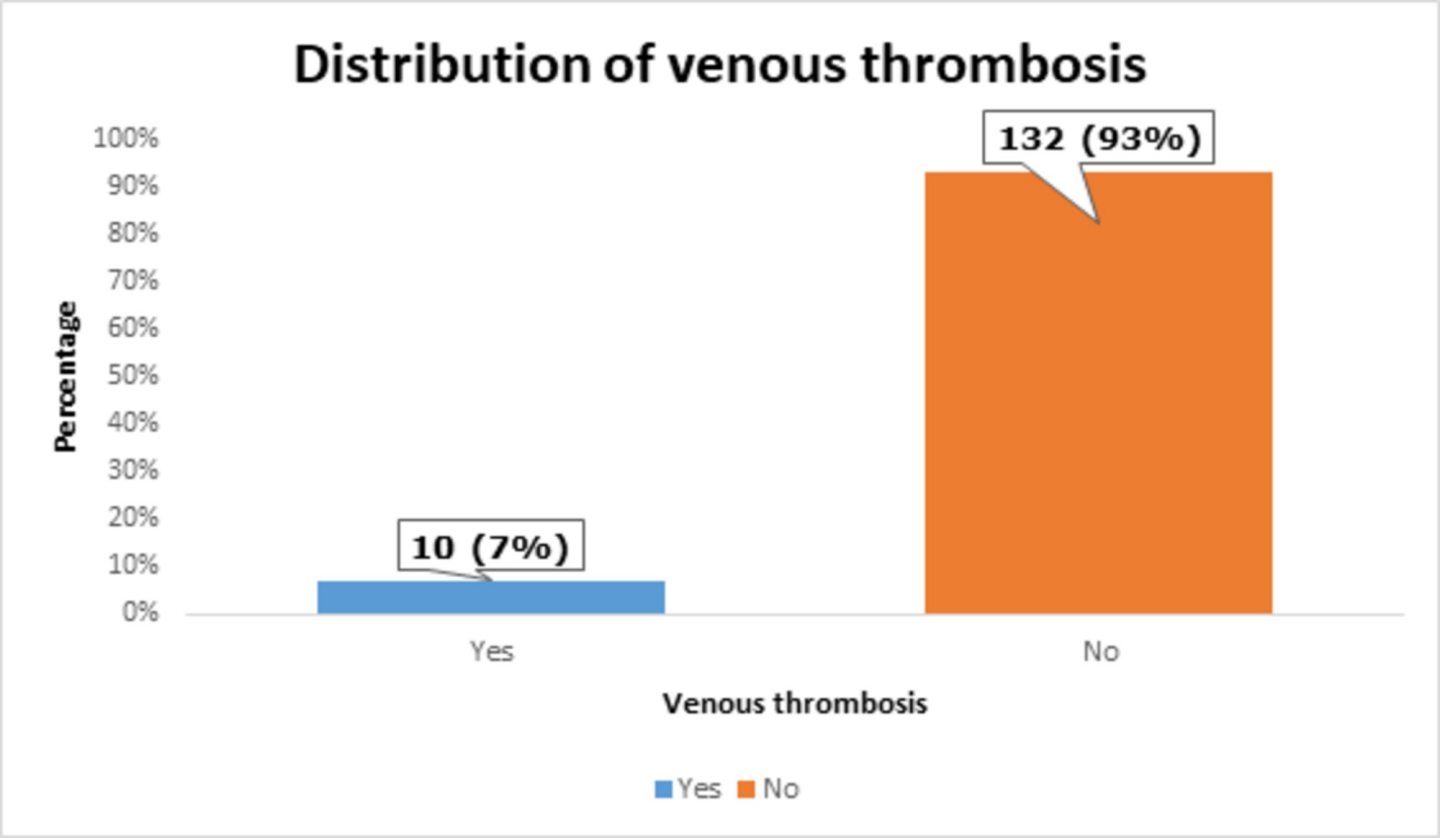
Distribution of venous thrombosis

Venous thrombosis was found in 10 (7%) of the patients, as shown in Figure 8.

Stratification of age, gender, obesity, Child-Pugh class, and duration of symptoms was done with venous thrombosis and a significant linear relationship was found with gender (p-value= 0.040), obesity (p-value= 0.043), and Child-Pugh class (p-value= 0.001), as shown in Table 6.

**Table 6:** Stratification of age, gender, obesity, duration of symptoms, and Child-Pugh class with venous thrombosis

Parameters		Venous thrombosis		p-value
		Yes	No	
Age (years)	≤60	2	45	0.497
	>60	8	87	
Gender	Male	7	46	0.040
	Female	3	86	
Obesity	Yes	9	72	0.043
	No	1	60	
Duration of symptoms (months)	≤6	4	82	0.192
	>6	6	50	
Child-Pugh class	A	1	85	0.001
	B	3	36	
	C	6	11	

## Discussion

We found in our study that there is a 7% frequency of venous thrombosis in cirrhotic patients, and it has a linear association with male gender, obesity, and a worsening Child-Pugh score.

Northup and associates studied hospitalized patients, including those with cirrhosis for a period of eight years, and concluded that cirrhotic patients had a reduced risk of venous thromboembolism, i.e., 0.5% as opposed to those admitted with other medical conditions. They further tried to find out which features were different in the cirrhotic patients who developed venous thromboembolism, i.e., 113 patients as compared to those who did not develop venous thromboembolism over this eight-year study period but could not see any major difference of the cause of cirrhosis or the other features such as mean international normalized ratio (INR), mean modified Model for End-stage Liver Disease (MELD) score, mean total bilirubin, mean serum creatinine, and mean platelet count between the two groups. The only significant difference between the two groups was their mean serum albumin concentration (a mean of 2.85 g/dL in cases versus 3.10 g/dL in controls and a p-value of < 0.01 was noted). Therefore, they concluded that a low serum albumin concentration can give us a clue of the possible increased risk of venous thromboembolism in cirrhotic patients and the likely explanation they gave was that severe liver dysfunction is associated with more decline in not only the serum albumin concentration but also anticoagulants like protein C, protein S, and anti-thrombin III. Despite the fact that the mean serum albumin concentration was considerably different between the two groups, the clinical use of this difference (0.25 g/dL) will be of little importance [5].

Gulley et al. also did an analysis in hospitalized patients, including 963 cirrhotic and 12,405 non-cirrhotic patients, with other medical illnesses. He found that although the mean international normalized ratio (INR) was higher in patients with liver cirrhosis as compared to other patients (a mean of 1.7 ± 2.1 in cases and 1.1 ± 0.7 in controls, and a p-value of <0.001 was noted) but even then, they were more prone to develop venous thromboembolism than patients with other diseases (1.87% in cases versus 0.98% in controls with a p-value of 0.007) except those with chronic kidney disease (7%), congestive heart failure (7.75%), and cancer (6.1%) with a p-value of <0.05. He also noted that the frequency of venous thromboembolism increased as the Child-Pugh score worsened (p-value = 0.10). Then, he performed a univariate analysis of all the patients and found that cirrhosis, the Charlson Index Score (usually higher in cirrhotic patients), hemoglobin concentration, INR, partial thromboplastin time (PTT), and serum albumin concentration were significantly associated with venous thromboembolism but on multivariate analysis, only two of these factors, i.e., low serum albumin and elevated PTT were related with venous thromboembolism [8].

Wanless et al. suggest that the probable mechanism in the development and progression of cirrhosis, due to any cause and especially congestive heart failure, is portal vein and hepatic vein thrombosis that leads to hypoxia, ischemia, and extinction of the liver parenchyma and eventually leads to fibrosis [10-11].

## Conclusions

The venous thromboembolism is not only a frequent complication in cirrhotic patients but also plays an important role in the pathogenesis and progression of cirrhosis. Therefore, it should be kept in mind while dealing with cirrhotic patients, especially those with male gender, obesity, and Child-Pugh class B or C, to prevent lethal consequences. The likely factors that can predict its occurrence are low serum albumin and increased PTT. But more analysis is needed on a larger scale to find out its exact frequency and prognostic tools to prevent it.
